# A feasibility study on yoga’s mechanism of action for chronic low back pain: psychological and neurophysiological changes, including global gene expression and DNA methylation, following a yoga intervention for chronic low back pain

**DOI:** 10.1186/s40814-022-01103-2

**Published:** 2022-07-07

**Authors:** Bandita Adhikari, Angela Starkweather, Wanli Xu, Rebecca L. Acabchuk, Divya Ramesh, Bright Eze, Yuxuan Yang, Gee Su Yang, Joseph Walker, Reinhard Laubenbacher, Crystal L. Park

**Affiliations:** 1 Penn Frontotemporal Degenerative Center, Philadelphia, PA USA; 2 Center for Advancement in Managing Pain, University of Connecticut School of Nursing, 231 Glenbrook Road, Storrs, CT 06269 USA; 3 Department of Psychological Sciences, University of Connecticut, Storrs, CT USA; 4 Senior Clinical Scientist, Apellis Pharmaceuticals, Boston, MA USA; 5 Department of Orthopedic Surgery and Neurosurgery, UConn Health, Farmington, CT USA; 6 Systems Medicine Lab, University of Florida, Gainesville, FL USA

**Keywords:** Chronic low back pain, Emotion regulation, Gene expression, Methylation, Yoga

## Abstract

**Introduction:**

Yoga has been shown to reduce pain and improve function in populations with chronic low back pain (cLBP), yet the underlying molecular mechanisms remain elusive. This study examined the feasibility and acceptability of a yoga research protocol, including recruitment, retention, and data collection, and investigated the preliminary effects of yoga on psychological and neurophysiological functions, including gene expression and DNA methylation profiles, in participants with cLBP.

**Methods:**

A one-arm trial was conducted with 11 participants with cLBP who enrolled in a 12-week yoga intervention. Data on subjective pain characteristics, quantitative sensory testing, and blood for analysis of differentially expressed genes and CpG methylation was collected prior to the start of the intervention and at study completion.

**Results:**

Based on pre-determined feasibility and acceptability criteria, the yoga intervention was found to be feasible and highly acceptable to participants. There was a reduction in pain severity, interference, and mechanical pain sensitivity post-yoga and an increase in emotion regulation and self-efficacy. No adverse reactions were reported. Differential expression analysis demonstrated that the yoga intervention induced increased expression of antisense genes, some of which serve as antisense to known pain genes. In addition, there were 33 differentially hypomethylated positions after yoga (log2 fold change ≥ 1), with enrichment of genes involved in NIK/NF-kB signaling, a major pathway that modulates immune function and inflammation.

**Discussion/conclusions:**

The study supports the feasibility and acceptability of the proposed protocol to test a specific mechanism of action for yoga in individuals with cLBP. These results also support the notion that yoga may operate through our identified psychological and neurophysiologic pathways to influence reduced pain severity and interference.

**Supplementary Information:**

The online version contains supplementary material available at 10.1186/s40814-022-01103-2.

## Key messages

What uncertainties existed regarding the feasibility?

This feasibility addressed the uncertainty of participant adherence to weekly yoga sessions and homework as well as the acceptability of collecting pain phenotype data, specifically through quantitative sensory testing and blood collection. In addition, the feasibility of analyzing changes in pain, gene expression, and DNA methylation profiles across time points was addressed.

What are the key findings on feasibility?

The protocol was found to be highly feasible and acceptable to participants, and preliminary analyses suggest a possible mechanism of action of yoga on pain by increasing self-efficacy and emotion regulation as well as through changes in gene expression and DNA methylation profiles.

What are the implications of the findings for the design of the main study?

The implications of the findings for the design of the main study include the capacity to identify changes in pain severity, self-efficacy, emotion regulation, and neurophysiological changes over the 12-week yoga intervention. The findings support the study protocol in terms of measures used, timing, and methods of analysis.

## Introduction

Chronic low back pain (cLBP) is the most prevalent chronic pain condition, afflicting up to 10% of adults at any given time and 70% of all people during their lifetime [1]. Most acute back pain episodes resolve within 4 weeks [2], but a third of those who seek treatment report persistent pain 1 year later [3–5]. Variability in defining chronicity of low back pain is common, but a useful definition of cLBP [6] is “low back pain lasting for 3 out of the last 6 months.” In addition to the pain itself, individuals with cLBP are at risk for symptoms and functional limitations including increased disability, anxiety, and depression and reduced quality of life [7–11]. As a leading cause of years lost to disability [12], cLBP is associated with millions of work absentee days and has been recognized as one of the most costly public health issues of the twenty-first century owing to its increasing prevalence [13], rising costs of healthcare expenditures, and association with opioid analgesic abuse [14, 15].

The etiology of cLBP is complex; both psychosocial and neurophysiological factors contribute to its development and persistence [16]. In particular, the biosignature of cLBP involves neurosensory alterations that manifest as enhanced pain sensitivity, functional neuroplasticity in pain processing centers of the brain, and altered expression of pain sensitivity genes and their receptors, suggesting a molecular basis in the “memory” of pain-facilitatory pathways [17–19].

Treatment recommendations for cLBP are directed by causal factors [20], yet most cases (85%) are nonspecific, or not linked to any specific physical or structural pathology [21]. Although a range of conventional pharmacologic, non-pharmacologic, and surgical procedures are used for non-specific cLBP, most patients report only modest or moderate relief [22], which is likely because most therapeutic strategies do not target the underlying mechanisms of cLBP. Commonly, management for non-specific cLBP includes advice to remain physically active, education on back self-care, medication, and physical therapy (PT) [20]; however, patient satisfaction with the effectiveness of conventional cLBP treatment is relatively low [23]. Thus, there is a substantial need for research to identify more helpful and acceptable therapies [24].

Yoga practice is a commonly used integrative therapy across the world, with reported use to improve health and relieve pain nearly doubling over the past decade in many countries [25]. A growing number of randomized controlled trials (RCTs) [26–33] support yoga’s effectiveness for reducing pain and improving function in adults with cLBP. Reviews, meta-analyses [34, 35], and practice guidelines from the American Pain Society and the American College of Physicians [20] support yoga as an evidence-based treatment for cLBP with at least moderate benefit. The prevalence of practicing yoga as an integrative mind and body therapy has increased, but the mechanisms of action (MOAs) by which it exerts clinical improvement on pain (severity and interference) remain unclear.

Several investigators have attempted to examine potential mediators of yoga [31, 36] on cLBP outcomes, but they have lacked a theoretical basis for assessment of the mechanism(s) involved. Most notably, Sherman et al. [29] conducted a 12-week RCT to examine the effect of Viniyoga versus conventional therapeutic exercise or a self-care book for patients with cLBP (*n* = 101). Although no group differences in pain emerged, pain bothersomeness decreased and function increased in the yoga group compared to the exercise and self-help book groups. Subsequently, Sherman et al. [30] conducted a 12-week RCT to examine the effect of yoga versus intensive stretching versus a self-help book (*n* = 228). Self-efficacy, hours of practice, and sleep quality were identified as mediators of function in the yoga group [36]. However, this study examined only a small set of potential mediators and explained a fairly small portion of the variance in improvement. Thus, although yoga has been shown to reduce pain and improve function in populations with cLBP, the MOAs remain to be elucidated. Importantly, yoga interventions for cLBP typically use a protocol based on a general understanding of yoga as a mind–body modality, with some effort to incorporate yoga postures that seem well-suited to improve functioning and decrease pain. However, these interventions have rarely attempted to optimize explicitly on MOAs, perhaps because they are yet to be identified.

In consideration of yoga’s MOA on cLBP, a wealth of data has shown that psychological factors contribute to cLBP [20, 37, 38]; thus, effectiveness of yoga may depend on the participant’s uptake of emotion regulation skills and ability to manage their pain (self-efficacy). More recent studies document functional alterations in people with cLBP that reflect peripheral and central nervous system sensitization, a condition referred to as enhanced pain sensitivity [17, 19]. As a mechanism of cLBP, enhanced pain sensitivity involves sensitization of nociceptors and neuronal circuits [18] and increased pain signaling through membrane excitability and synaptic efficacy [39], which are conferred through altered expression of pain-sensitivity genes [40]. Immune function, specifically inflammatory responses to tissue injury and coordination of healing, also contributes to pain modulation and sensory alterations [41, 42]. The somatosensory changes associated with enhanced pain sensitivity in cLBP involve decreased thermal and mechanical pain thresholds at the site of pain and remote areas [19]. Studies evaluating dysfunctional conditioned pain modulation in cLBP have not demonstrated consistent results [43, 44]; however, the possibility that psychological factors and insufficient descending pain controls play a role in cLBP has been of high interest [45]. Of importance, physical activity is associated with reduced psychological risk factors and pain sensitivity [46]. Thus, there are several potential MOAs involving psychological and neurophysiologic pathways by which yoga may improve cLBP outcomes.

Of particular interest, several studies of yoga-based interventions have demonstrated an effect on upregulating genes associated with energy metabolism, mitochondrial function, insulin secretion, telomere maintenance [47–50], and downregulating expression of genes linked to inflammatory response and stress-related pathways [51–53]. Our research has shown that there are specific clusters of genes dysregulated during the transition from acute to cLBP and that the pattern of dysregulated gene expression is associated with differential DNA methylation between individuals who either report resolution of their pain or a cLBP trajectory [54]. Thus, another plausible MOA of yoga for cLBP may be through modification of epigenetic (methylation) and gene expression profiles. Noting the importance of coordinating participant engagement and data collection of the proposed MOA, a feasibility study was conducted to inform the methods of a larger clinical trial designed to identify the psychological and neurophysiologic MOA of yoga for individuals with cLBP.

## Specific aims

The primary aim of this study was to examine the feasibility (recruitment, adherence to weekly sessions and homework) and acceptability of the yoga intervention protocol. The secondary aim was to explore the pre- to post-test changes in the proposed MOAs (self-efficacy, emotion regulation, pain sensitivity, gene expression, and methylation) as proof of concept for a larger investigation.

## Material and methods

A one-arm feasibility study was conducted to assess the research protocol and provide proof of concept for the proposed MOA. Our approach to the design and reporting of this feasibility study was informed by Thabane et al.’s [55] adaptation of the CONSORT statement. The study protocol was approved by the University of Connecticut’s Institutional Review Board.

### Participants and setting

Men and women between the ages of 18 and 70 years of age diagnosed with nonspecific cLBP and able to read and write in English were invited to participate through emails sent to existing volunteers of our affiliated Center’s contact registry and through advertisements targeting college campuses and the general community. This age range was selected to provide a more homogeneous sample in terms of general health, work status, and contributing factors of cLBP.

Nonspecific cLBP was defined as pain without a specific cause or need for surgical intervention that was anywhere in the region of the low back bound superiorly by the thoraco-lumbar junction and inferiorly by the lumbo-sacral junction, which had been present for ≥ 3 months out of the prior 6 months and was self-rated at a current level of ≥ 4 on the numeric rating scale (NRS). Recruitment took place at an urban university health system after approval from the Institution Review Board. All participants provided written consent prior to study participation.

Potential subjects with cLBP were excluded for the following conditions: (a) pain at another site or associated with a painful condition (e.g., degenerative disc disease, herniated lumbar disc, fibromyalgia, neuropathy, rheumatoid arthritis, sciatica); (b) previous spinal surgery; (c) presence of neurological deficits; (d) history of comorbidities that affect sensorimotor function (e.g., multiple sclerosis, spinal cord injury); (e) pregnant or within 3 months postpartum; (f) taking opioid, or anticonvulsant medication; (g) history of psychological disorders (e.g., bipolar disorder, schizophrenia) because of possible associations with pain genomic pathways; and (h) already meeting the US Department of Health and Human Services, Office of Disease Prevention and Health Promotion physical activity recommendations of 150 min per week of moderate-intensity aerobic activity or already using yoga once per week or greater.

### Sample size

The study was designed to assess the feasibility and acceptability of the intervention protocol as well as determine the preliminary efficacy signal of the intervention, specifically, whether the intervention alters the proposed MOA. Preliminary efficacy was evaluated by comparing the pre- to post-test results for pain severity, self-efficacy, emotional regulation, and gene expression and methylation profiles. The target sample size of *n* = 10 was based on a pragmatic approach for assessment of feasibility in the study process, resources, and management as recommended [56, 57] with specific criteria for success of feasibility listed in Table 1.

**Table 1:** Research protocol feasibility and acceptability outcomes

**Feasibility outcomes**	
**Recruitment**	The goal was to recruit 10 participants over a 2-week period who were willing to commit to the intervention schedule. The screen-to-enroll ratio was determined, which will be used to estimate the number of volunteers needed to screen for a full-scale trial to achieve the desired sample size
**Adherence**	The goal was for all participants to complete at least 8 out of the 12 intervention sessions (≥ 65%) and the homework, which was based on a prior large-scale trial.^30^
**Retention**	The goal was to retain 80% of participants and complete all outcome assessments within 1 week of the participant’s last session. The retention rate was determined and will inform the attrition estimation and plan for over-sampling in the full-scale trial
**Acceptability outcomes**	
Participant experience with the study	We conducted quantitative and qualitative assessments of the participant experience with the intervention and research protocol to assess the need for modifications. Participants were asked what they liked and did not like about the intervention and research protocol, what if anything they noticed changed during the intervention, and what they would change about the intervention or research protocol in the future
Participant satisfaction	Participants completed a satisfaction questionnaire after they completed the study

### Procedures

After obtaining a history and physical examination to identify exclusion criteria, participants were scheduled to undergo baseline data collection as soon as possible but no longer than 1 week from the time of consent. Data collection took place in a private research suite at the study site. Participants were asked to complete demographic questions about their age, gender, socioeconomic status, educational attainment, lifestyle behaviors (smoking, exercise), comorbidities, past episodes of LBP, and current cLBP characteristics (severity and interference). Following completion of the questionnaires, participants underwent venipuncture for the collection of blood samples and quantitative sensory testing (QST). The sequence of data collection was followed for all participants. The same sequence of data collection (questionnaires, venipuncture, and QST) was carried out at the 12-week visit.

The yoga sessions took place each week at a university gymnasium with ample room for participants to perform poses. Participants were given small incentives; a yoga mat at the first yoga session and a $25 gift card to a local store that has both food and personal items at the completion of the second study session. Following completion of the 12-week intervention, interviews were conducted by a research team member to evaluate participant experiences and satisfaction with the study protocol.

#### Description of the yoga for cLBP intervention

The yoga intervention was led by a certified instructor who specializes in working with individuals with cLBP. The yoga intervention protocol relied on classical Hatha yoga with influences from Viniyoga and Iyengar yoga. Both Viniyoga and Iyengar yoga styles emphasize modifications and adaptations including the use of props such as straps and blocks in order to minimize the risk of injury and make the poses accessible to people with health problems and limitations. The certified yoga instructor led participants through a manualized series of 23 yoga poses (32 total variations) at a slow-moderate pace.

Yoga classes were constructed to allow optimal flow from one pose to another. Each session began with a few minutes of deep breathing and mindfulness/meditation followed by about 15 min of basic postures (poses 1–8) to warm up muscles by increasing circulation to provide increasing flexibility as the class progressed. Most poses were conducted once per side or with the opposite foot leading. After warm up, the instructor led participants through a series of standing poses (poses 9–14) for about 15 min. After the standing poses, the class moved into floor poses (poses 15–22) for about 20 min. Sessions ended with 5–7 min of complete relaxation in the standard ending pose “savasana” during which additional positive affirmations were provided along with a body scan. During the poses, the instructor cued for ujjayi breathing and integrated cues for mindfulness, acceptance, nonjudgement, and self-compassion throughout the practice. Participants were encouraged to emulate optimal alignment as demonstrated by the instructor and to focus on a goal or positive direction for their yoga practice.

The importance of attending all sessions was emphasized during the first session and throughout all subsequent sessions and participants received regular email and text reminders to attend. To facilitate their home practice, the yoga instructor and research team provided participants with a written guide on developing a yoga practice routine that included links to online yoga videos, a booklet on managing low back pain, and an 18-min video created by the yoga instructor. Participants were encouraged to perform home practice for 10–15 min on days they were not attending the in-person classes, and to not push themselves when practicing at home or to stop home practice if their pain or discomfort was noticeable. In addition, participants received email prompts every 48 h to fill out their practice logs, which were administered online through REDCap, a secure web application for building and managing online surveys and databases.

### Measures

#### Demographics and general questionnaires

A brief 40-item questionnaire based on the minimum dataset as recommended by the NIH Taskforce on Research Standards for cLBP [6] was used to assess demographics (age, gender, race/ethnicity, education, socioeconomic and employment status) and medical history (duration of LBP, comorbidities, medication usage, ongoing treatments). Height, weight, and body mass index were measured using a calibrated floor scale and height rod.

#### Pain severity and interference

The Brief Pain Inventory (BPI) is a pain assessment tool that has well-established reliability and validity for adult patients with LBP and sensitivity to change over time [58]. The BPI assesses the severity of pain, location of pain, pain medications, amount of pain relief in the past 24 h and the past week, and the impact of pain on daily functions.

#### Pain self-efficacy

Self-efficacy for managing pain reflects confidence in the person’s ability to influence the intensity or impact of LBP on daily life. The pain self-efficacy measure consisted of 8 items rated from 1 (very uncertain) to 10 (very certain) and summed [59]. Developed originally for arthritis pain, it has been adapted for studies of cLBP and has demonstrated good psychometrics.

#### Emotion regulation

The Emotion Regulation Questionnaire (ERQ) [60] assesses two *specific emotion regulation strategies, suppression and reappraisal*. The ERQ comprises 10 items (5 for suppression and 5 for reappraisal) rated from 1 (never [do this] to 7 [always do this]). The ERQ has demonstrated strong psychometric properties [60].

#### Attendance/home practice

Attendance of yoga sessions was assessed using sign-in logs verified by the yoga instructor. Self-reported practice of yoga at home was assessed using a weekly participant yoga log. The yoga log documented the amount of time practiced, the use of instructions, the difficulty of poses, estimated level of exertion, and any adverse events such as increased pain.

#### Satisfaction with the yoga class

After the completion of the yoga class, a 17-item instrument was used to assess participants’ satisfaction with the yoga class and suggestions for improvement. The instrument was developed for this study and asked questions about perceived benefits and challenges of participating in yoga and intention to continue using yoga.

#### Quantitative sensory testing

QST uses standardized stimuli to evaluate both nociceptive and non-nociceptive systems in response to mechanical and thermal stimuli [61]. QST was performed on the lumbar region and the dominant forearm (remote area). A standardized protocol of administration, including examination room conditions and instructions provided for the participant, was strictly followed from the same protocol described in the preliminary analysis reported by our group [19, 62]. Participants were given a practice run on the non-dominant forearm in order to verify the participant’s understanding of the protocol.

#### Molecular biological measures

##### Genomic DNA isolation and quality control

Genomic DNA (gDNA) was extracted from the white blood cell layer using standard protocols (Qiagen, DNA mini kit). Genomic DNA was quantified and purity ratios were determined for each sample using the NanoDrop 2000 spectrophotometer (Thermo Fisher Scientific, Waltham, MA, USA). To further assess DNA quality, samples were analyzed on the Agilent TapeStation 2200 (Agilent Technologies, Santa Clara, CA, USA) using the Genomic DNA assay. DNA Integrity Numbers (DIN) were recorded for each sample, and all were ≥ 4.0 for library preparation.

### Reduced representation bisulfite sequencing (RRBS-seq) sample preparation

Genomic DNA samples (400 ng input target) were prepared for Illumina-compatible library preparation based on the previously published methodology [63]. In summary, samples were digested overnight at 37 °C with the restriction enzyme MspI (New England Biolabs, Ipswich, MA) in 50μL total volume. Digested DNA was purified using QIAquick PCR purification columns (Qiagen, Hilden, Germany) in preparation for end repair. Reagents for end repair, A-tailing, and adapter ligation were sourced from the Illumina TruSeq DNA Nano sample preparation kit (Illumina, San Diego, CA); protocol for end repair, A-tailing, and adapter ligation followed Morison et al. [63]. Adapted library molecules were size selected using the Pippin Prep on a 2% agarose cassette with external markers for selection of 160–340 bp (Sage Science, Beverly MA), then purified using QIAquick PCR purification columns (Qiagen, Hilden, Germany). Bisulfite conversion was completed using the EZ DNA Methylation-Gold kit (Zymo Research, Irvine CA). Samples were bisulfite converted overnight following the manufacturer’s recommended protocol. Size selected and bisulfite converted samples were PCR amplified and purified following Morison et al. [63]. Final libraries were validated for length and adapter dimer removal using the Agilent TapeStation 2200 D1000 High Sensitivity assay (Agilent Technologies, Santa Clara, CA, USA) then quantified and normalized using the dsDNA High Sensitivity Assay for Qubit 3.0 (Life Technologies, Carlsbad, CA, USA). Sample libraries were prepared for NextSeq 550 sequencing using version 2 sequencing chemistry (paired end 2 × 75 bp read length).

#### Pilot study outcomes

##### Feasibility and acceptability outcomes

Feasibility outcomes included recruitment, adherence to weekly sessions, and completion of homework, as well as retention with completion of an outcome assessment battery. Acceptability was assessed using mixed methods: acceptability and satisfaction questionnaires and semi-structured interviews at the end of the study. The a priori anticipated outcomes are described in Table 1.

##### Exploratory assessment of the yoga intervention’s efficacy

Pre- and post-intervention study questionnaires were informed by the IMMPACT guidelines on outcomes relevant to chronic pain clinical trials and included the acceptability assessments described below [64].

### Statistical analysis

Normal distribution of the data was initially assessed using SPSS software version 24. No imputations were made for missing data. Outcomes were reported as means and standard deviations (SDs) for continuous variables and frequencies and percentages for categorical variables. For continuous variables, *t*-tests were used to compare pre- to post-test scores; however, in keeping aligned with the objectives of a feasibility study, only the 95% confidence intervals are reported. Interviews were analyzed thematically by two independent coders (RA, AS). Discrepancies were reconciled by a third study team member (DR). Representative quotes are presented for themes that helped us understand why and how the intervention could work.

#### RNA-seq analysis

RNA-sequencing was performed on an Illumina NextSeq 550 System, and FASTQ files were generated after removing barcodes and adapters. Raw reads from FASTQ files were then processed to generate read counts for gene alignment. In brief, the reads were checked for quality using FastQC v 0.11.7 (https://www.bioinformatics.babraham.ac.uk/projects/fastqc) and trimmed using TrimGalore v 0.6.5 for any adapters that may still be present. Reads with a Phred score lower than 28 were discarded. FastQC and TrimGalore were repeated until desired reads were obtained. MultiQC v.1.7 was used to combine individual FastQC results for visualization. The high-quality cleaned reads were then aligned to the Ensembl GrCh38v99 reference genome using STAR 2.7.2b, and Qualimap 2.2.1 was used to check the quality of alignment. Ensembl GRCh38v99 reference genome (sequence.fa files) and annotation (.gtf files) were downloaded from Ensembl. Read-count matrix was created with the reads from the fourth column of the readspergene.tab STAR output files for reverse strandedness using our in-house Python script. DESeq2 v1.29, an R package, was used to perform differential expression analysis between the pre-yoga and post-yoga intervention. DESeq2 Negative Binomial GLM fitting and Wald statistics were used to obtain differentially expressed (DE) genes. The resulting *p*-values were adjusted for false discovery using Benjamini and Hochberg’s approach, and genes with an adjusted *p*-value < 0.1 were considered as differentially expressed. We used a 0.1 threshold for significance because this study was exploratory and the goal was to prevent losing interesting genes by using a stricter threshold. Plots were generated using R packages, EnhancedVolcano, and ggplot2. Gene enrichment analysis was performed with the differentially expressed genes (*p*-value < 0.1) using the Database for Annotation, Visualization and Integrated Discovery (DAVID) analysis.

#### RRBS-seq analysis

RRBS-sequencing was performed on an Illumina NextSeq 550 System. Quality control for RRBS reads was performed in a similar fashion to RNA-seq. Raw reads were checked for quality using FastQC v 0.11.7 and trimmed using TrimGalore v 0.6.5. Reads shorter than 20 bp and lower than Phred score 20 were discarded. The trimmed and cleaned reads were then aligned to the Ensembl GrCh38v99 reference genome using Bismark (–score_min L, 0, -0.4). Methylated CpGs were extracted using R package- EdgeR. The *edgeR* function readBismark2DGE was used to collate the counts from bismark.cov coverage files for all samples into one data object. Any genomic segments that were not assembled into any of the known chromosomes were discarded. CpG sites with at least a total count of 8 for methylated and unmethylated combined in all 16 samples were used for further analysis. The reads were normalized using the library size. EdgeR was used to perform differential methylation analysis between groups pre- and post-yoga intervention. We fit Negative Binomial Generalized Linear Models for all the CpG loci using the glmFit() function, and glmLRT() was used to perform likelihood ratio tests to obtain differential methylation. Differential methylation at each CpG, promoter level, and chromosome levels for both groups were studied. Gene enrichment analysis was performed with genes associated with differentially methylated positions (adjusted *p*-value < 0.1) using David Ontology. GO plot and ggplot2 were used to create plots for data visualization.

## Results

### Baseline participant characteristics and pre-post data

**Fig. 1:**
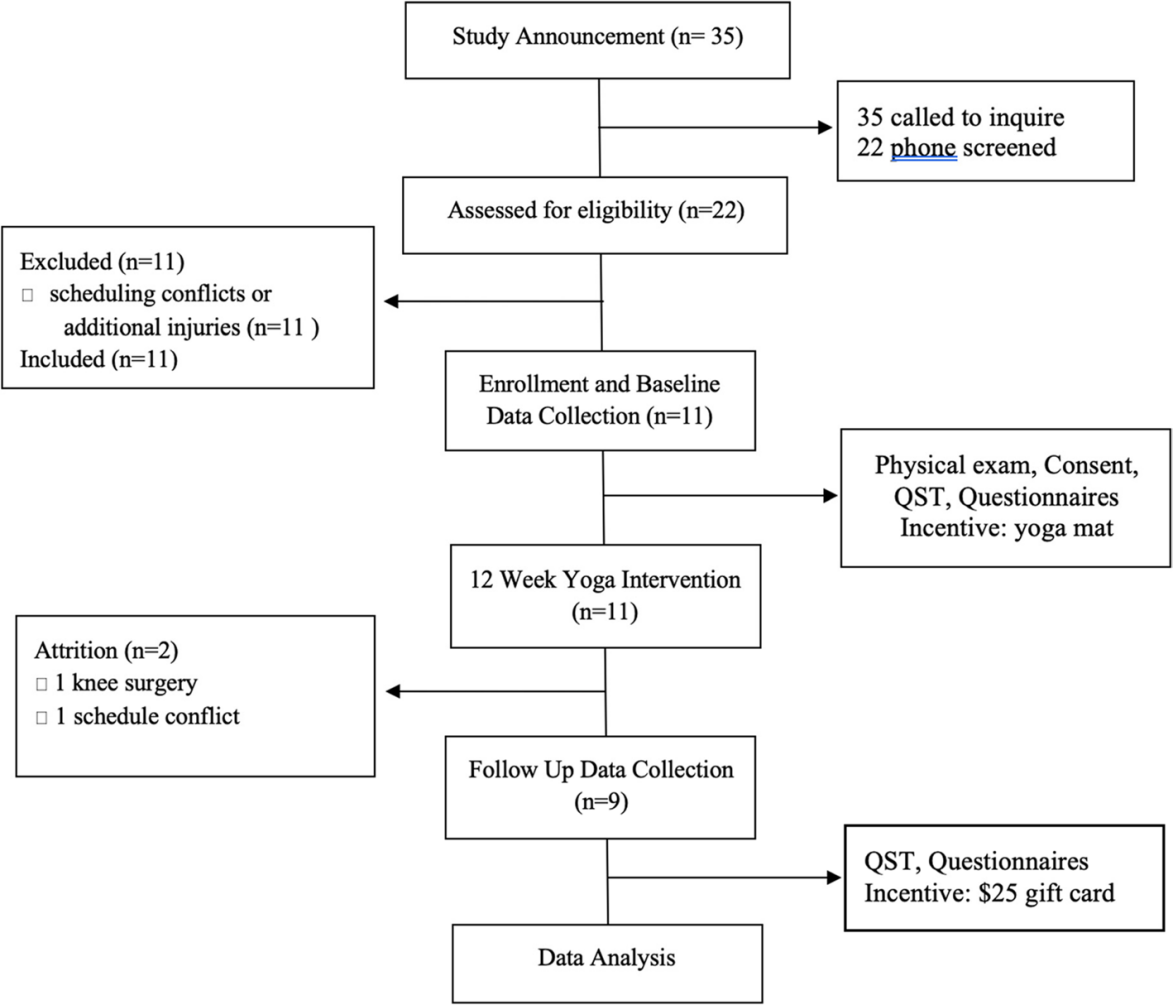
Participant flow: CONSORT diagram

The final study sample included 11 participants, 1 male and 10 females, with a mean age of 34 years (range 19–64 years, SD = 15.8). Figure 1 details the participant flow through the study. The mean duration of cLBP was 3.4 years (SD = 3.02). There were 1 Asian and 10 White/Caucasian participants. None of the participants was taking opioid analgesics, and 2 participants reported intermittent ibuprofen use for pain management. Relative to baseline, participants reported lower pain severity and interference, and greater self-efficacy and emotion regulation-suppression score after the yoga intervention (Table 2). The effect size for change in mean scores was large for pain severity and interference (0.97), medium for self-efficacy (0.79), and small for emotion regulation — suppression (0.36). Quantitative sensory testing measures were z-transformed according to values provided by pain-free normal controls collected by the laboratory (Table 3).

**Table 2:** Psychosocial and neurophysiological measures

Construct	Measure	T1 mean (SD)	T2 mean (SD)	95% CI (lower, upper)
Pain severity	BPI SF – pain now	2.9 (2.3)	0.9 (0.9)	0.57, 3.48
	BPI SF – average	3.8 (1.4)	2.1 (1.6)	0.30, 3.11
	BPI SF – worst pain	5.4 (2.1)	2.3 (1.7)	1.29, 4.77
	BPI SF – least pain	1.27 (1.42)	0.44 (.5)	− 0.06, 1.71
Pain interference	BPI SF – pain interference	17.5 (14.4)	6.3 (8.0)	− 0.9, 22.51
Pain self-efficacy	Pain self-efficacy	6.1 (2.4)	7.7 (1.8)	− 3.09, − 0.08
Emotion regulation	ERQ reappraisal	29.9 (8.3)	29.4 (3.4)	− 3.77, 4.69
	ERQ suppression	15.9 (7.9)	13 (7.9)	− 0.31, 4.31
Pain sensitivity	Mechanical pain sensitivity – control	1.6 (1.6)	1.7 (1.1)	− 1.42, 1.22
	Mechanical pain sensitivity – pain site	2.6 (2.0)	1.4 (.65)	− 0.14, 2.48
	Cold Pain Threshold – control	17.2 (8.7)	17.1 (7.7)	− 6.47, 6.59
	Cold Pain Threshold – pain site	13.3 (8.0)	10.3 (2.9)	− 1.09, 7.07
	Heat Pain Threshold – control	40.9 (3.6)	41.4 (3.4)	− 2.76, 1.87
	Heat Pain Threshold – pain site	41.1 (2.7)	42.0 (3.2)	− 2.36, 0.68
	Pressure Pain Threshold – control	248.3 (109.6)	276.5 (97.5)	− 84.15, 27.74
	Pressure Pain Threshold – pain site	256.3 (80.4)	283.3 (96.6)	− 93.69, 39.69

*BPI SF* Brief Pain Inventory Short Form, *ERQ* Emotion Regulation Questionnaire

**Table 3:** QST Z-scores comparing yoga participants and 11 matched pain-free controls

Measure	T1 mean (SD)	T2 mean (SD)	95% CI (lower, upper)
Mechanical pain threshold – control	− 4.10 (4.99)	− 1.45 (2.22)	− 7.29, − 0.90
Mechanical pain threshold – pain site	− 1.53 (1.41)	− 0.63 (1.36)	− 2.61, − 0.44
Mechanical pain sensitivity – control	0.43 (1.16)	0.5 (0.83)	− 0.53, 1.39
Mechanical pain sensitivity – pain site	0.43 (1.08)	− 0.19 (0.34)	− 0.49, 1.36
Cold Pain Threshold – control	0.79 (1.12)	0.78 (0.99)	− 0.16, 1.74
Cold Pain Threshold – pain site	0.72 (0.97)	0.36 (1.17)	− 0.16, 1.60
Heat Pain Threshold – control	− 0.98 (1.51)	− 0.40 (0.85)	− 2.12, 0.17
Heat Pain Threshold – pain site	− 0.83 (1.09)	− 0.49 (1.30)	− 1.80, 0.10
Pressure Pain Threshold – control	− 0.64 (0.95)	− 0.40 (0.85)	− 1.53, 0.25
Pressure Pain Threshold – pain site	− 1.25 (0.73)	− 1.00 (0.88)	− 2.05, − 0.46

### Changes in gene expression and DNA methylation

#### Yoga therapy induces expression of antisense genes in chronic low back pain

**Fig. 2:**
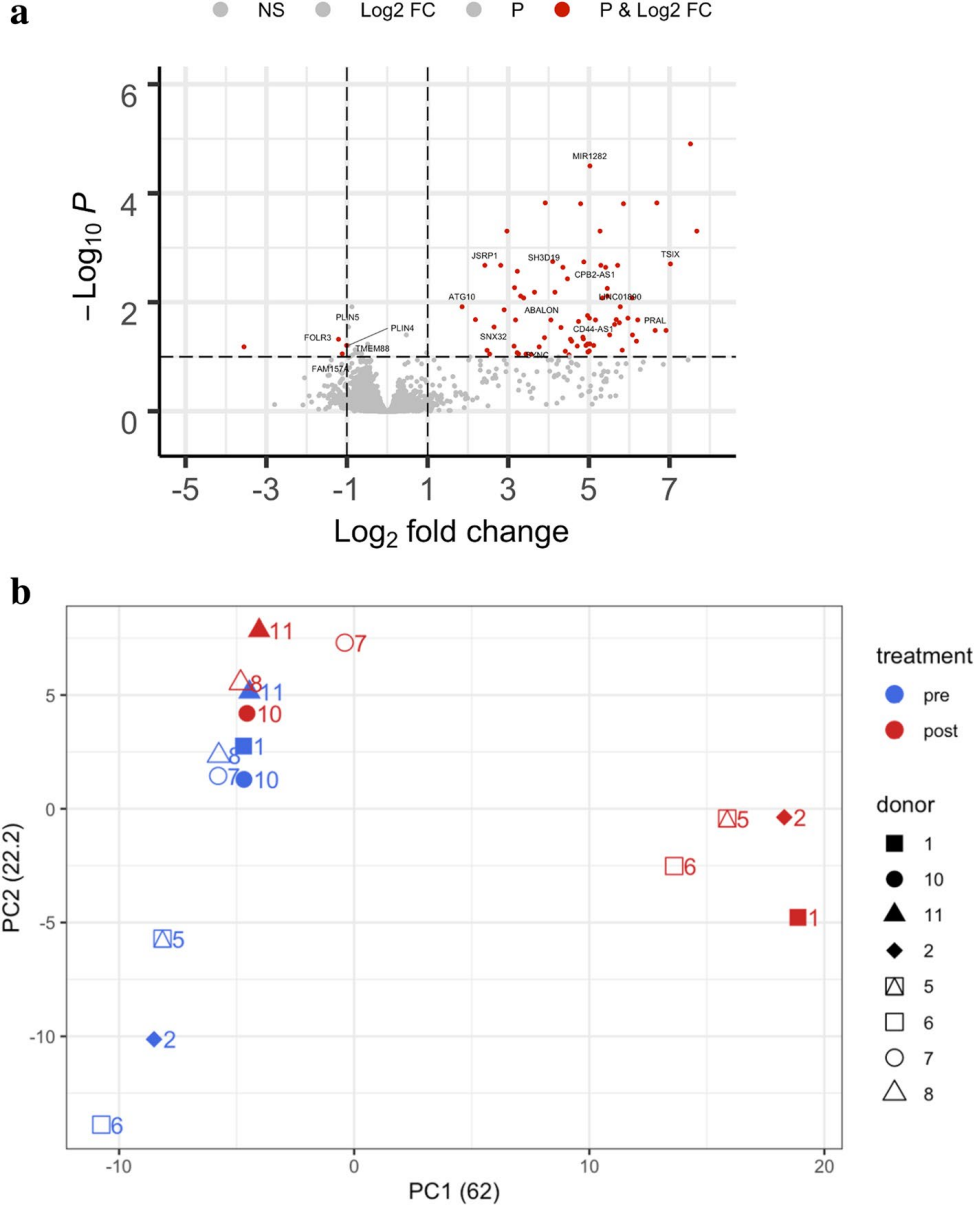
**a** Volcano plot. Volcano plots comparing Log2 (fold change) of read count values for the post-yoga group vs the pre-yoga group. Significantly upregulated and downregulated genes at the threshold of (adj pval < 0.1, log2FC > 1) are shown in red. **b** PCA plot of top 500 DE genes. PCA plots showing overall differences in expression between the samples. Principal components were computed for top 500 differentially expressed genes using prcomp() R function and top two PCs are plotted. Four replicate samples (1, 2, 5, 6) from the Post group cluster together. The first dimension, PC1, separates the four Post samples from the rest of the samples

A total of 94 genes were reported as differentially expressed by DESeq2 at significance of alpha of 0.1, of which 76 genes were upregulated and 18 were downregulated. The results are visually represented on a volcano plot (Fig. 2a) with statistical significance (negative log of adjusted *P*-value) on the *y*-axis versus the magnitude of change (Log2 fold change) on the *x*-axis. The top 50 DE genes were used for Ingenuity Pathway Analysis and DAVID analysis, but the genes did not belong to any specific biological process or pathway. We discovered that most of the reported DE genes did not have associated HGNC gene symbols. Only 36 out of 94 genes had assigned gene names; others belong to different categories of processed transcripts: antisense (29), pseudogenes (5), sense (1), and novel transcripts (23) (Supplemental Table 1).

We further studied the antisense transcripts and their connection to pain since natural antisense transcripts are known to play a role in regulating human pain perceptions and chronic pain disorders [65]. While the exact mechanisms of regulation by antisense RNA transcripts are not fully understood yet, they are believed to do so by interfering with their expression, RNA editing, RNA masking, RNAi, and chromatin modifications [66, 67]. Some of the reported differentially expressed antisense transcripts were antisense to genes that have previously been reported to be relevant to pain conditions. Specifically, killer cell lectin-like receptor K1 (*KLRK1*), karyopherin subunit beta 1 (*KPNB1*), and lysosomal protein transmembrane 5 (*LAPTM5*), as well as intercellular adhesion molecule-3 (*ICAM-3*), sphinolipin, ribosomal protein L23a (*RPL23A*), and synaptosome-associated protein 23 (*SNAP-23*), are pain-related genes to which the differentially expressed processed transcripts were antisense [68].

#### Molecular changes in expression due to yoga is associated with better pain outcomes

We further looked into the relationship between the samples using principal component analysis (PCA) of the top 500 DE genes ranked by adjusted *p*-value. Figure 2b shows that post-intervention samples 1,2,5, and 6 form a separate cluster than the rest of the subjects. In the PCA plot, the PC1 component, which corresponds to the yoga treatment explains 62% of variation in the data and mainly contributes to the differential gene expression observed in our analysis. Variable loadings that reflect the weight that each gene has on principal components PC1 and PC2 are available in Supplemental Table 2. The other samples of the post-yoga group (7, 8, 10, 11) were closer to the pre-yoga group indicating that yoga treatment did not affect these subjects at the transcriptional level. Samples 1, 2, 5, and 6 also had better pain outcomes via lower pain sensitivity and self-reported pain scores after yoga treatment compared to other samples in the same group (mean reduction in worst pain was 3.0 v 1.5 and mean reduction in pain “now” was 2.25 v 0.5). The analysis suggests that the yoga intervention is effective in 50% of the test population, and most importantly, the effectiveness in this population seems to be associated with changes at the transcriptional level.

#### Hypomethylation of genes involved in NIK/NF-KB signaling following yoga therapy

**Fig. 3:**
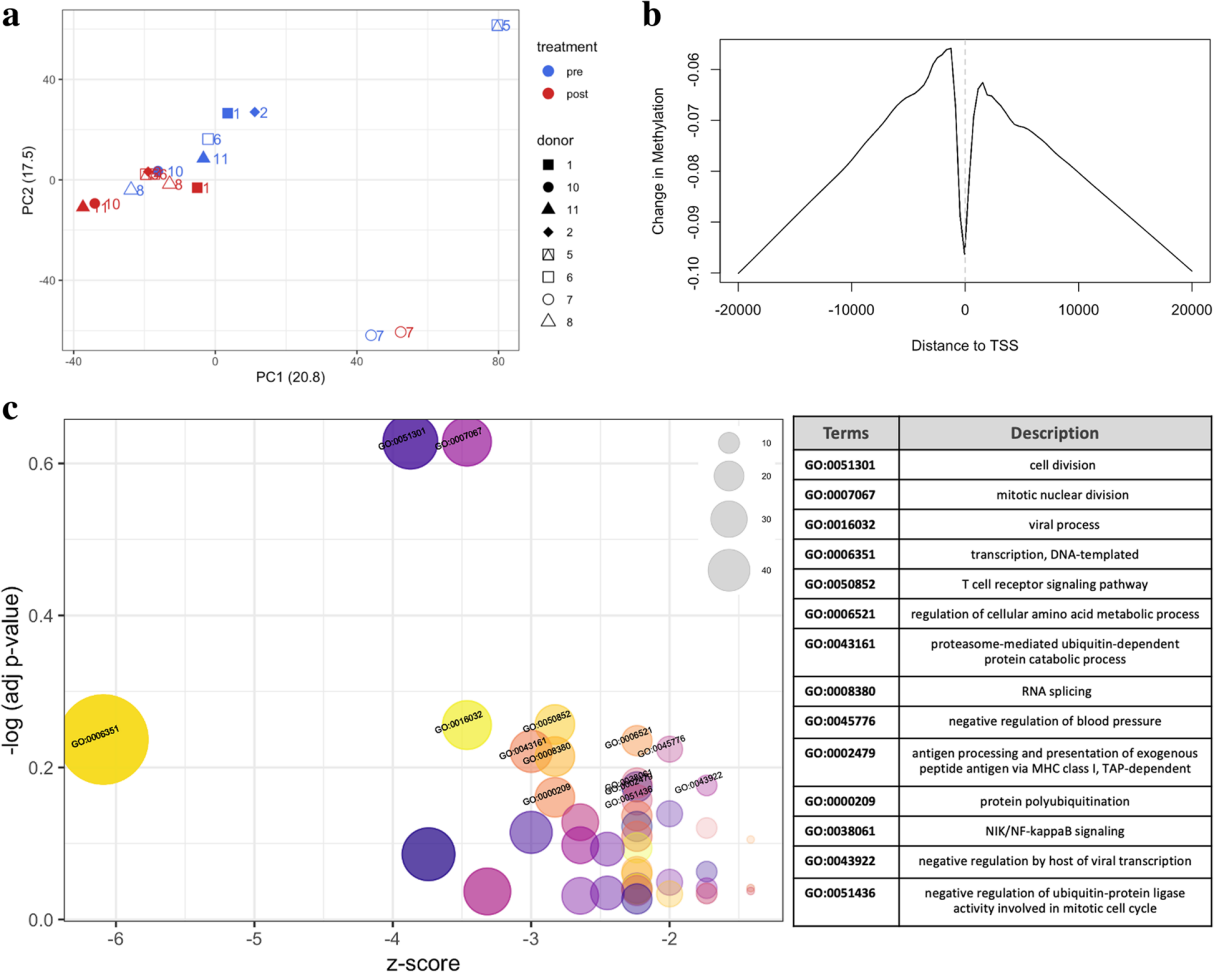
**a** PCA plot of top 500 DE methylated genes. PCA plots showing overall differences in methylation levels between the samples. Principal components were computed for top 500 methylated promoters using prcomp() function and top two PCs are plotted. Post and Pre groups do not form distinct separate cluster. **b** Change in methylation in the Post vs Pre group. Average methylation change in the post-yoga group vs pre-yoga group by genomic position relative to TSS. Changes are averaged over all genes. Negative and postive distances indicate the upstream and downstream regions of TSS. The vertical axis shows base 2 logit differences. **c** Top DE methylated genes DAVID ontology. Gene ontology analysis for top differentially methylated genes

To determine if there were changes in DNA methylation patterns acquired from the yoga intervention, we examined individual CpG sites, gene promoters, and chromosomes. Given the promoter of a gene is located within a 3-kb region, and 2 kb upstream to 1 kb downstream from the transcription start site of that gene, promoter regions were annotated with the gene names for analysis. The top 500 differentially methylated promoter data was used to plot the relationship between all samples (Fig. 3a). The PCA plot shows a minimal difference between the post- vs pre-yoga samples.

However, the differential methylation analysis showed a total of 282 with 5 hypermethylated and 277 hypomethylated positions (Benjamini-Hochberg-adjusted *p*-value of 0.1). Out of 282, only 33 positions are differentially methylated by twofold (log2FC ≥ 1), and all of them are hypomethylated. Reduced methylation has also been reported previously in women suffering from chronic stress who underwent yoga treatment [69]. Consistent with this, Fig. 3b shows the overall change in methylation in the post-yoga intervention group versus the pre-yoga. The post-yoga group has lower methylation than the pre-yoga groups, and the methylation is lower around the TSS in both groups, and CpGs located further from the TSS are unchanged.

With the identified genes associated with differentially methylated positions, we performed a DAVID gene ontology (GO) analysis (Fig. 3c). The IDs associated with GO terms on the plot are shown in the legend table. The most significant biological processes were “cell division” and “mitotic nuclear division.” Many genes are also enriched for “transcription, DNA templated.” One of the pathways shown to be implicated in pain is nuclear factor-kappa B (NF-κB) inducing kinase (NIK/NF-κB) signaling. In our dataset, NIK/NF-κB signaling appears as an enriched pathway (*p*-value = 0.01, padj = 0.6). Specifically, 5 genes, such as proteasome 26S subunit, non-ATPase 1 (*PSMD1*), proteasome 26S subunit, non-ATPase 12 (*PSMD12*), proteasome subunit alpha type-3 (*PSMA3*), proteasome subunit beta type-6 (*PSMB6*), and proteasome subunit beta type-10 (*PSMB10*), in NIK/NF-κB signaling were hypomethylated in the post-yoga samples.

### Pilot study outcomes

#### Recruitment and enrollment

Recruitment and enrollment took place over a 2-week period, with 35 people responding immediately to a single announcement, and over 50% of those screened eligible to participate.

#### Adherence and retention

Two participants were lost to attrition (due to knee surgery and schedule conflict). All remaining participants adhered to study requirements: they attended at least 8 group yoga sessions offered over 12 weeks, established a home practice, and completed the daily yoga log following at least 30 out of 40 prompts that were emailed to them every 48 h for the duration of the study.

### Acceptability

#### Participant experience and satisfaction

Based on self-report follow-up questionnaires administered at the final intake, participants reported an overall satisfaction level of 90% (9 average score rating on a 10-point scale, SD = 1.1). No adverse events, such as increased pain or injuries, were reported over the study duration. Participants reported improved physical movement, reduced stress levels, and reduction in pain, and 100% of participants planned to continue yoga practice to help manage their cLBP.

The yoga classes have had a significant impact on my life, and have helped my body to be stronger, more flexible and pain-free.

Seventy-five percent of participants reported feeling a change in relationship to their body or pain.

Awareness of alignment and posture [lead to a] significant reduction in lower back pain.

I can control the pain through movement/stretching, loosening, releasing muscle tension to relieve pain.

62.5% reported needing to take less analgesics since starting yoga.

I learned how to acknowledge my sensations in my back without necessarily running to medication.

One hundred percent of participants reported that the tools to practice at home were useful.

The video was most helpful. The tools served as a reminder to do home practice.

The most common obstacles for developing a home practice were time and having a quiet space for practice, but all participants reported developing strategies to overcome these obstacles. Participants reported the following as the most helpful aspects of yoga class: asanas (seated, standing and supine poses) (*n* = 4), alignment (*n* = 4), flexibility (*n* = 3), breathing techniques (*n* = 2), poses for strength (*n* = 2), balance (*n* = 2), and centering (*n* = 1).

I thought it was a good combination of poses, very gentle, excellent for beginners.

Using a 10-point scale, participants rated the areas of greatest benefit from yoga. Top ranking results included improved physical movement (*M* = 8.4, *SD* = 1.6), reduced stress levels (*M* = 8.1, *SD* = 1.8), and reduction in pain (*M* = 7.6, *SD* = 2.4).

The back lengthening giving more space between the vertebrae. The breathing relaxed the muscles but the alignment and flexibility poses helped with whatever caused the pain. It relaxed my mind away from stress.

Perceived mechanisms of benefits included improvements in calm mindset (specifically from pranayama/breathwork) (*n* = 6), mindfulness/acceptance (*n* = 5), self-awareness (*n* = 4), attitudes towards stress (*n* = 4), coping mechanisms (*n* = 4), and spiritual well-being (*n* = 1).

Breathing exercises helped with body awareness and esteem, coping with stress is easier.

Participants reported positive feedback about the instructor, stating the instructor was “very knowledgeable/helpful,” “positive,” and “made us feel at ease.”

I liked that she [the instructor] explained why these things helped our back, and made sure to help with proper pose posture.

To further understand the neurophysiologic changes that are activated in response to yoga in individuals with cLBP, we analyzed differential mRNA and DNA methylation pre/post-intervention in blood samples. The analytic pipeline was completed to identify genes and CpG sites of interest that could help guide further analysis in larger participant samples.

## Discussion

This study examined the feasibility and acceptability of the yoga intervention protocol and investigated the preliminary effects of yoga on psychological and neurophysiological measures, including gene expression and DNA methylation profiles, in cLBP. The feasibility criteria were met for the target sample size and adherence to intervention attendance in sessions and homework. Although attrition was higher than anticipated, this was due to extraneous circumstances as satisfaction with the research and intervention protocol was high and intention to continue yoga beyond the study was notable. Following the yoga intervention, pain severity and interference, pain self-efficacy, and emotion regulation improved. According to a multicenter study, most individuals with cLBP report poor self-efficacy and specifically, individuals who rely solely on pharmacological interventions are more likely to have low self-efficacy [68]. Given that low self-efficacy can be a challenge to the management of cLBP, our result supports the notion that yoga is beneficial as one of the psychological rewards in managing cLBP as evidenced by prior research [27, 70, 71] and the themes that emerged from the qualitative analysis of this study.

Analysis of DE genes from pre- to post-intervention identified a unique transcriptional response to yoga, and these participants reported a greater reduction in pain following the yoga intervention. Antisense transcripts of several genes, such as *KLRK1*, *KPNB1*,* LAPTM5*,* ICAM-3*, and *RPL23A*, were most notable in this group of responders. *KLRK1* is an activator of NK cell cytotoxicity, and NK cells have been shown to have a greater cytotoxic activity in individuals with cLBP [72] as well as spondyloarthritis [73]. Similarly, *KPNB1* is a key regulator of NF-κB signaling in chronic peripheral neuropathic pain [74]. In addition, *LAPTM5* has been found to be crucial for neuropathic pain in chronic constriction injury (CCI) animal models [75]. *ICAM-3* and *RPL23A* have also been reported to be dysregulated in rheumatoid arthritis synovia [76, 77]. An understanding of the role of antisense RNA transcripts may help discover novel therapeutic biomarkers for treating cLBP [78, 79]. As we mentioned above, the majority of genes associated with hypomethylated positions were enriched for “DNA templated transcription” and possibly this could be linked to an increased expression of these antisense RNA transcripts.

Given that “cell division” and “mitotic nuclear division” seem the most significant biological processes in the analysis of genes with methylated positions, the cell cycle pathway could be implicated in epigenetic alterations in the interaction with yoga on pain. It is well acknowledged that epigenetic modifiers (e.g., DNA methylation, histone modification, chromatin structure alteration) can modulate cell cycle progression by activating or silencing cell cycle-related genes or by segregating chromosomes and condensing chromatin so that the new daughter cells have the correct epigenetic inheritance [80]. Prior research suggested that the regulation of the cell cycle may be associated with the development and maintenance of pain. Wu and colleagues found that cell cycle inhibition reduces mechanical and thermal hyperalgesia as well as locomotor dysfunction via glial changes in spinal cord injury pain and the cell cycle pathways can mediate the contribution of the truncated isoform of the brain-derived neurotrophic factor (BDNF) receptor tropomysin-related kinase B. T1 (trkB.T1) to neuropathic pain in spinal cord injury in animal models [81, 82]. Future studies may be required to examine its epigenetic role in regulating cell cycle pathways in cLBP.

Further analysis of differential methylation from pre- to post-yoga blood samples found 33 genes associated with hypomethylated positions by twofold (log2FC ≥ 1), with enrichment of genes involved in NIK/NF-κB signaling. NF-κB can be activated through the canonical pathway via stimuli that include tumor necrosis factor (TNF)-alpha, interleukin (IL)-1, and toll-like receptors, or the noncanonical pathway through NIK [83]. The NIK pathway is activated by B cell-activating factor (BAF) which is a TNF family receptor, receptor activator of NF-κB (RANK), and lymphotoxin (LT) [84]. Activation of NF-κB is detected primarily in glutamatergic neurons and can protect neurons through regulation of neuronal inflammatory reactions and surrounding neuronal environment [85]. Nociceptive A-delta and C-fibers that comprise the axons of dorsal root ganglion neurons are glutamatergic, thereby providing a direct pathway to influence pain processing [84], as demonstrated by decreased generalized pain sensitivity from pre- to post-yoga.

Of the genes associated with differentially hypomethylated positions that are involved in NIK/NF-κB signaling, *PSMD1* and *PSMD12* are protein-coding genes that play a key role in protein homeostasis and DNA damage repair. These proteasomes and others are components of the ubiquitin–proteasome system (UPS) and cellular protein quality control (PQC), with the UPS playing a role in inflammatory responses as regulators of leukocyte proliferation [80]. Proteasomes are responsible for activating NF-κB, which in turn, regulates the expression of pain facilitatory molecules, including proinflammatory cytokines, adhesion molecules, prostaglandins, and nitric oxide, which have shown variable reduction after yoga and other mind–body interventions [86–88]. *PSMA3* is required for the generation of a subset of MHC class I-presenting antigenic peptides (20S – PA28 complex) while *PSMB6* and *PSMB10* also display peptidylglutamyl-hydrolizing activity to clear acidic residues. In our DE gene analysis, *KPNB1* is also a key regulator of NF-κB signaling, and genes associated with hypomethylation of the NIK/NF-κB signaling pathway lend support although preliminary for a role of NF-κB as a MOA of yoga-induced pain reduction in individuals with cLBP.

This study has several limitations. We have a small sample size of one cohort with no controls due to its intended nature as a feasibility study, which limits the generalizability of the results. In addition, the principal component analysis (PCA) should be interpreted with caution as PCA typically requires at least 5 observations per variable included. These results must be replicated in larger samples and the investigative team is currently working on this endeavor. However, the present analysis provides an initial focus for future large-scale multi-omic research on the MOA of yoga for cLBP. Another limitation is that the participants were not required to be fasting at the time of their blood draw which could have resulted in variation of the expression profiles, and candidate genes and hypomethylated positions identified in this study require further analysis to confirm biological pathways. Several top-ranked differentially expressed genes and CpG sites can be validated by the quantification of mRNAs using RT-PCR and the examination of long interspersed element-1 (LINE-1) methylation along with other epigenetic modifications. Lastly, we were not able to determine the association between changes in methylation ratios and changes in pain outcomes due to the small sample size; however, a regression model would be used to see directional associations adjusting for other confounding factors in a larger study.

## Conclusions

This pilot study demonstrated the feasibility and acceptability of the yoga intervention, with all of participants planning to continue yoga practice to help manage cLBP. The yoga intervention improved self-reported physical movement, reduced stress levels, reduced pain severity, and interference as well as improved pain self-efficacy and emotion regulation. Our results may provide new insights into the influence of yoga on genetic and epigenetic mechanisms in cLBP. Participants with greater pain severity reduction demonstrated differential neurophysiologic responses following the yoga intervention, which supports the notion that such biobehavioral analyses could be used to tailor yoga and possibly other mind–body therapies for pain management.

## Supplementary Information

**Additional file 1:** **Supplemental Table 1.** Differentially expressed (non-HGNC) processed transcripts.

**Additional file 2:** **Supplemental Table 2.** Variable loadings of PCA (RNA-seq data).

## Data Availability

Researchers interested in the data that supports the study findings should contact the corresponding author once appropriate Institutional Review Board and Data Agreement and Sharing approval have been obtained.
