# Supplementary material for: A feasibility study on yoga’s mechanism of action for chronic low back pain: psychological and neurophysiological changes, including global gene expression and DNA methylation, following a yoga intervention for chronic low back pain
Source: Pilot Feasibility Stud. 2022 Jul 7;8:142. doi: 10.1186/s40814-022-01103-2 (PMC9260994; doi:10.1186/s40814-022-01103-2)
Supplement: Supplementary file 1 — Additional file 1: Supplemental Table 1. Differentially expressed (non-HGNC) processed transcripts. [file 40814_2022_1103_MOESM1_ESM.docx]

Supplemental Table 1: Differentially expressed (non-HGNC) processed transcripts
