# Supplementary material for: A feasibility study on yoga’s mechanism of action for chronic low back pain: psychological and neurophysiological changes, including global gene expression and DNA methylation, following a yoga intervention for chronic low back pain
Source: Pilot Feasibility Stud. 2022 Jul 7;8:142. doi: 10.1186/s40814-022-01103-2 (PMC9260994; doi:10.1186/s40814-022-01103-2)
Supplement: Supplementary file 2 — Additional file 2: Supplemental Table 2. Variable loadings of PCA (RNA-seq data). [file 40814_2022_1103_MOESM2_ESM.docx]

Supplemental Table 2: Variable loadings of PCA (RNA-seq data)
